# Racial And Ethnic Disparities In Patient Experience Of Care Among Nonelderly Medicaid Managed Care Enrollees

**DOI:** 10.1377/hlthaff.2021.01331

**Published:** 2022-02

**Authors:** Kevin H. Nguyen, Ira B. Wilson, Anya R. Wallack, Amal N. Trivedi

**Affiliations:** Brown University, Providence, Rhode Island.; Brown University.; University of Vermont Health Network, Burlington, Vermont.; Brown University and Providence Veterans Affairs Medical Center, Providence, Rhode Island.

## Abstract

Medicaid managed care enrollees who are members of racial and ethnic minority groups have historically reported worse care experiences than White enrollees. Few recent studies have identified disparities within and between Medicaid managed care plans. Using 2014–18 data on 242,274 nonelderly Medicaid managed care enrollees in thirty-seven states, we examined racial and ethnic disparities in four patient experience metrics. Compared with White enrollees, minority enrollees reported significantly worse care experiences. Overall adjusted disparities for Black enrollees ranged between 1.5 and 4.5 percentage points; 1.6–3.9 percentage points for Hispanic or Latino enrollees; and 9.0–17.4 percentage points for Asian American, Native Hawaiian, or other Pacific Islander enrollees. Disparities were largely attributable to worse experiences by race or ethnicity within the same plan. For all outcomes, disparities were smaller in plans with the highest percentages of Hispanic or Latino enrollees, and for some outcomes, there were smaller disparities in plans with the highest percentages of Asian American, Native Hawaiian, or other Pacific Islander enrollees. Interventions to mitigate racial and ethnic inequities in care experiences include collection of comprehensive race and ethnicity data, adoption of health equity performance metrics, plan-level enrollee engagement, and multisectoral initiatives to dismantle structural racism.

Managed care has become the dominant care delivery model for Medicaid enrollees, with nearly 70 percent of beneficiaries enrolled in a comprehensive managed care plan in 2018.^1^ To hold Medicaid managed care plans accountable for care provided to enrollees, most states collect experience-of-care metrics and use them in state quality improvement initiatives, such as value-based purchasing and public reporting.^1^ Despite tremendous growth in the number of Medicaid managed care enrollees nationwide, particularly in states that extended Medicaid eligibility through the Affordable Care Act (ACA),^2^ there is limited recent evidence regarding patient experience among Medicaid managed care enrollees.^3,4^

Medicaid plays a key role in serving low-income racial and ethnic minority populations—approximately half of nonelderly Medicaid beneficiaries are members of racial and ethnic minority groups.^5^ Some evidence suggests that Medicaid managed care enrollees are satisfied with their health care, although minority enrollees report having worse experiences.^6,7^ Well-documented and longstanding racial and ethnic disparities in patient experience are the product of interwoven patient-, provider-, and plan-level factors, as well as systemic inequality. Structural racism, which refers to the ways in which racial discrimination is infused into policies and social norms through mutually reinforcing systems (for example, health care and housing), is a driver of worse experiences of care for minority populations.^8^ Interpersonal racism (for example, biases and discrimination), differential expectations of care, availability of culturally inclusive services, and patient-provider concordance (such as by race and ethnicity, sex, or language) also affect patients’ access to and experiences of care.^9–11^ For example, Medicaid enrollees who experience racial or ethnic discrimination are significantly less likely to report getting needed care or timely access to specialty care.^12^ Conversely, patient-provider language concordance and availability of professional interpreter services are associated with greater satisfaction.^11^

To better inform strategies that mitigate racial and ethnic disparities in experience of care among Medicaid managed care enrollees, it is critical to determine whether variation is primarily due to within-plan or between-plan disparities. Within-plan disparities measure differences in patient experience between non-Hispanic White and racial and ethnic minority patients enrolled in the same plan. Between-plan disparities occur when minority patients are disproportionately enrolled in plans that deliver low-quality care for all racial and ethnic groups. This distinction has important implications for how such disparities are addressed. We assess racial and ethnic disparities in four experience-of-care metrics among Medicaid managed care enrollees and estimate the extent to which overall disparities maybe attributed to within-plan disparities versus between-plan disparities.

## Study Data And Methods

### DATA

We pooled five years of data from the National Committee for Quality Assurance (NCQA) Adult Medicaid Consumer Assessment of Healthcare Providers and Systems (CAHPS) Health Plan Survey, version 5.0 (2014–18). CAHPS surveys are enrollee-level surveys that assess the patient experience of care. They are submitted annually to the NCQA by state Medicaid agencies and individual Medicaid managed care plans. Adult Medicaid CAHPS sampling was limited to people who were age eighteen or older, reported their primary coverage through a Medicaid managed care plan, and were continuously enrolled for the previous six months. Survey modes include telephone, mail, and internet, and all data are collected by a NCQA-certified third-party vendor. The CAHPS survey domains collect data on enrollee demographics and health status, personal doctors, specialists, health plan experience, and health care in the previous six months. We merged CAHPS data with Medicaid managed care enrollment reports, which are submitted annually by states to the Centers for Medicare and Medicaid Services (CMS) and estimate total plan enrollees as of July 1 of each year.^13^

### STUDY SAMPLE

Our study sample included nonelderly adults (ages 18–64) enrolled in a Medicaid managed care plan between 2014 and 2018 in thirty-seven states offering comprehensive Medicaid managed care plans during our study period. We excluded a small proportion (3.5 percent) of enrollees in prepaid inpatient or ambulatory health plans, limited benefit plans, and plans that primarily served children or people dually eligible for Medicare and Medicaid. We also excluded enrollees of plans located in Puerto Rico, enrollees ages sixty-five and older, enrollees missing age data, and respondents who did not report a race or ethnicity (online appendix exhibit 1).^14^ Because submission of CAHPS data to the NCQA is not mandatory, our sample was limited to plans that reported these data.

### MEASURES

Our main explanatory variable was enrollee self-reported race and ethnicity. In the CAHPS survey, enrollees were asked whether they were of Hispanic or Latino origin or descent. Race was measured using six categories: White, Black or African American, Asian American, Native Hawaiian or other Pacific Islander, American Indian or Alaska Native, or other race. Our analyses focused on four mutually exclusive groups: Hispanic or Latino (any enrollee of Hispanic or Latino origin or descent, regardless of race); non-Hispanic White; non-Hispanic Black; and non-Hispanic Asian American, Native Hawaiian, or other Pacific Islander. We refer to enrollees in these four categories as Hispanic or Latino; White; Black; and Asian American, Native Hawaiian, or other Pacific Islander. Although they are included in our study sample, our main analysis did not include estimates for American Indian or Alaska Native enrollees (because of small sample size), multiracial enrollees, and enrollees who report other race; we report these populations in appendix exhibit 9.^14^

The study outcomes were four experience-of-care measures: whether an enrollee answered that it was “always or usually” easy to get needed care, that they had a personal doctor, that they were “always or usually” able to get a checkup or routine care as soon as they needed to, or that they were “always or usually” able to see a specialist as soon as they needed to. Definitions built on recent work using Medicaid CAHPS data.^6^ Survey items and responses are in appendix exhibit 2.^14^

### STATISTICAL ANALYSIS

We used Pearson’s chi-square tests to assess racial and ethnic disparities in sociodemographic characteristics and self-reported health status. We also calculated the average percentage of enrollees from each racial and ethnic group across the plans in our sample. Building on prior work, we fit linear probability models to examine racial and ethnic disparities in patient experience of care, using White enrollees as the reference group, and differences were measured in percentage-point terms.^6,15^ We constructed three models. Model 1 adjusted for sociodemographic characteristics (age, sex, highest level of education attained), self-reported health status (excellent, very good, good, fair, poor), survey mode (mail versus telephone or internet), survey language, receipt of assistance in completing the survey (which included reading questions to the respondent, writing down answers given, answering questions for the respondent, translating questions into the respondent’s language, or other assistance), and year fixed effects. Model 2, which measured overall disparities, added state fixed effects. To examine within-plan disparities in outcomes, model 3 added plan fixed effects. Building on prior work, between-plan disparities were measured as the difference between overall disparities (model 2 estimates) and within-plan disparities (model 3 estimates).^3,16^ To adjust for potential within-plan correlations, we clustered standard errors at the plan level. Analyses were weighted by a plan’s annual total Medicaid enrollment to account for plan size.^17^ All analyses used Stata, version 15.

### SENSITIVITY ANALYSES

We present characteristics and estimates separately for Asian American and Native Hawaiian or other Pacific Islander enrollees;^7,18^ by survey language for Hispanic or Latino enrollees; by receipt of assistance completing the survey for Asian American, Native Hawaiian, or other Pacific Islander enrollees; and by receipt of translational assistance for Hispanic or Latino, other race, and Asian American, Native Hawaiian, or other Pacific Islander enrollees. We assessed the robustness of our estimates by fitting multilevel models with plan-level random effects;^19^ by including enrollees ages sixty-five and older and those missing age or race or ethnicity information; by excluding American Indian or Alaska Native, other race, or multiracial enrollees from our analysis; and by respecifying our three outcomes with Likert scales to only top-box responses (“always,” rather than “usually or always”). To test whether there was geographic variation in disparities, we reran our main analyses and included an interaction between race and ethnicity and census region (Northeast, South, Midwest, and South), using the Northeast as the reference group.

We also illustrated between-plan effects based on percentage of enrollment by each racial and ethnic minority group. First, we divided plans into quintiles based on the percentage of enrollees from each racial and ethnic minority group and graphed outcomes for White and minority enrollees across quintiles. Second, we graphed the relationship between unadjusted plan-level racial and ethnic disparities and percentage of enrollees from each minority group. To quantify this relationship, we calculated the sample correlation between measures using Pearson’s correlation coefficients.

### LIMITATIONS

Our study had several limitations. First, a small percentage of respondents did not report a race or ethnicity or age and therefore were excluded. As described above, we reran our analyses including enrollees missing race, ethnicity, or age data. Estimates were robust (appendix exhibits 8 and 15).^14^ Second, because of small sample sizes, we did not report estimates in the main analysis for American Indian or Alaska Native enrollees or enrollees reporting other race. Third, expectations of care may result in differences in responses across racial and ethnic groups and should be considered in interpreting our findings. Fourth, although some studies have suggested that there are differential patterns of patient experience of care from patients who have limited English proficiency, we were unable to produce similar estimates of heterogeneity in our data because there was no indicator for preferred language.^4,20^ Instead we conducted stratified analyses by survey language among Hispanic or Latino enrollees and by receipt of assistance completing surveys (including assistance translating) among Asian American, Native Hawaiian, or other Pacific Islander enrollees (appendix exhibits 11 and 12).^14^

Fifth, consistent with prior work, we did not test for the statistical significance of between-plan disparities in our main analysis.^16^ To provide additional insights on between-plan disparities, we included supplemental analyses (shown in appendix exhibits 19–24).^14^ Sixth, it is possible that our study sample was not representative of all nonelderly Medicaid managed care enrollees—however, the plans in our sample accounted for 66 percent of all Medicaid managed care plan enrollment nationally in the most recent year of data.^13^ Seventh, survey nonrespondents may have worse care experiences than respondents, so it is possible that our results overestimate care experiences.^21^ Although the response rate for the NCQA Adult Medicaid CAHPS Health Plan Survey was lower than that for some federal surveys, such as the American Community Survey, it was favorable relative to other national surveys of patient experience and access to care, such as the Health Reform Monitoring Survey.^22^

## Study Results

### CHARACTERISTICS OF ENROLLEES

The average plan-level response rate was 23.3 percent. Our study sample consisted of 242,274 nonelderly Medicaid managed care plan enrollees, of whom 49.9 percent were White; 21.6 percent were Black; 14.2 percent were Hispanic or Latino; 3.9 percent were Asian American, Native Hawaiian, or other Pacific Islander (exhibit 1); 0.6 percent were American Indian or Alaska Native; 1.8 percent self-reported other race; and 8.0 percent were multiracial (appendix exhibit 3).^14^ There were statistically significant racial and ethnic variations in sociodemographic characteristics and self-reported health status (exhibit 1). In appendix exhibits 3–6^14^ we present characteristics for enrollees who were American Indian or Alaska Native, other race, or multiracial, as well as other stratified analyses.

### OVERALL DISPARITIES

In unadjusted models, racial and ethnic minority enrollees reported significantly worse experiences than White enrollees for all four outcomes (exhibit 2). Overall adjusted disparities between White and Black enrollees ranged between −1.5 percentage points for access to needed care and −4.5 percentage points for access to a personal doctor (exhibit 3). The magnitude of disparity between White and Hispanic or Latino enrollees ranged between −1.6 percentage points for access to needed care and −3.9 percentage points for timely access to a checkup or routine care. Disparities were largest between White and Asian American, Native Hawaiian, or other Pacific Islander enrollees, ranging between −9.0 percentage points for access to a personal doctor and −17.4 percentage points for timely access to specialty care. For Black enrollees, adjusted estimates were similar in magnitude to unadjusted estimates, although generally attenuated (appendix exhibit 8).^14^ For Hispanic or Latino and Asian American, Native Hawaiian, or other Pacific Islander enrollees, the magnitude of disparity attenuated on adjustment for sociodemographic characteristics and state fixed effects (model 2) and when plan fixed effects were added to the model (model 3) (appendix exhibit 8).^14^ We also present estimates for American Indian or Alaska Native enrollees, multiracial enrollees, and enrollees reporting other race (appendix exhibit 9);^14^ Asian American and Native Hawaiian or other Pacific Islander enrollees (appendix exhibit 10);^14^ Asian American, Native Hawaiian, or other Pacific Islander enrollees by receipt of assistance (appendix exhibit 11);^14^ Hispanic or Latino enrollees by survey language (appendix exhibit 12);^14^ and Asian American, Native Hawaiian, or other Pacific Islander, Hispanic or Latino, and other race enrollees by receipt of translation assistance (appendix exhibit 13).^14^ Estimates using multilevel models, including enrollees ages sixty-five and older and enrollees who were missing age or race and ethnicity information and excluding American Indian or Alaska Native, other race, or multiracial enrollees, were similar in magnitude to results from our main analysis (appendix exhibits 14–16).^14^ Notably, enrollees missing race and ethnicity information reported significantly worse care experiences than White enrollees (appendix exhibit 15).^14^ When we respecified outcomes to use top-box scores, statistically significant White and Hispanic or Latino and White and Asian American, Native Hawaiian, or other Pacific Islander disparities remained, and the magnitudes of disparity generally were larger (appendix exhibit 17)^14^ than estimates in our main analysis (appendix exhibit 8).^14^ In contrast, Black enrollees reported care experiences comparable in magnitude to or significantly better than those of White enrollees. For all outcomes except access to a personal doctor, the magnitude of disparities between White and Hispanic or Latino enrollees in the West were smaller than in the Northeast (appendix exhibit 18).^14^

### WITHIN-PLAN DISPARITIES

Compared with White enrollees, Black enrollees in the same plan consistently reported worse experiences of care, ranging between −1.2 percentage points for access to needed care and −4.5 percentage points for access to a personal doctor (exhibit 3). There were significant within-plan disparities between White and Hispanic or Latino enrollees for all outcomes except access to needed care. Compared with White enrollees, Asian American, Native Hawaiian, or other Pacific Islander enrollees within the same plan reported significantly worse experiences of care on all metrics, ranging between −8.6 percentage points for access to a personal doctor and −16.8 percentage points for timely access to specialty care.

### BETWEEN-PLAN DISPARITIES

Between-plan disparities were relatively lower across racial and ethnic minority enrollees in comparison to within-plan disparities (exhibit 3). Consistent with prior work, we did not test for statistical significance of between-plan disparities in our main analysis,^16^ but we provide additional insights in appendix exhibits 19–24.^14^ There were not substantial between-plan disparities among White and Black enrollees, as suggested by the similar magnitude of White and Black disparities across quintiles (appendix exhibit 19)^14^ and weak, positive correlations between plan-level percentage of Black enrollees and plan-level disparities in performance (appendix exhibit 20).^14^ However, the magnitude of White and Hispanic or Latino disparities decreased across quintiles (appendix exhibit 21),^14^ and there were modest, positive correlations between plan-level percentage of Hispanic or Latino enrollees and plan-level disparities in performance on all four outcomes, ranging between 0.23 for access to a personal doctor and 0.44 for access to needed care (appendix exhibit 22).^14^ Similarly, the magnitude of White and Asian American, Native Hawaiian, or other Pacific Islander disparities decreased across quintiles for some outcomes (appendix exhibit 23), and there were modest positive correlations between plan-level percentage of Asian American, Native Hawaiian, or other Pacific Islander enrollees and plan-level performance on all outcomes (appendix exhibit 24).^14^

For some outcomes, attenuated disparities reflected comparable experiences by racial and ethnic minorities across quintiles and worse experiences among White enrollees in plans with the highest concentration of a racial or ethnic minority group; for example, the percentage of Asian American, Native Hawaiian, or other Pacific Islander enrollees reporting timely access to checkup or routine care was relatively similar across quintiles—however, White enrollees in plans with the lowest percentage of Asian American, Native Hawaiian, or other Pacific Islander enrollees reported higher levels of access than White enrollees in plans with the highest percentage of Asian American, Native Hawaiian, or other Pacific Islander enrollees (appendix exhibit 23, panel C).^14^ For other outcomes, attenuated disparities reflected better experiences by racial and ethnic minorities in plans with a high percentage of minority enrollees—for example, Hispanic or Latino enrollees reported higher levels of access to needed care than White enrollees in plans with the highest percentage of Hispanic or Latino enrollees (appendix exhibit 21, panel A)^14^ while reporting worse access to needed care than White enrollees in plans with the lowest percentage of Hispanic or Latino enrollees.

## Discussion

This is the first study to our knowledge to assess patient experience of care in a multistate sample of Medicaid managed care enrollees and to compare within- and between-plan disparities after the ACA.^3 4^ Consistent with prior work,^7^ we found that compared with White enrollees, racial and ethnic minority enrollees reported significantly worse experiences on all four metrics, and disparities were primarily driven by different experiences of care for minority enrollees within the same plan.^3^ These estimates are comparable with disparities in perceived quality of care in a national survey of low-income adult patients with other forms of insurance (Medicare or commercially insured) and disparities in care coordination among Medicare Advantage patients.^23,24^ For all four outcomes, plans in the highest quintile of Hispanic or Latino beneficiaries had smaller magnitudes of disparities for these enrollees than plans in the lowest quintile. These findings build on prior work in Medicare Advantage suggesting that contracts with higher percentages of Hispanic or Latino beneficiaries have smaller White and Hispanic or Latino within-contract disparities than those with lower percentages of Hispanic or Latino beneficiaries.^25,26^ Similarly, there were smaller magnitudes of disparities between the lowest and highest quintiles of Asian American, Native Hawaiian, or other Pacific Islander enrollment for some outcomes. It is plausible that plans with higher enrollment of Hispanic or Latino or Asian American, Native Hawaiian, or other Pacific Islander enrollees are able to provide more targeted engagement or outreach or to develop provider networks that perform better for these enrollees. Although we were unable to examine the mechanisms driving these differences in our data, these results warrant further exploration about what state- and plan-level strategies may lead to more equitable patient experience of care.

Medicaid managed care plans are uniquely positioned to address racial and ethnic disparities in patient experience of care. Many states use plan contracts as a primary lever to address such disparities in Medicaid. Identifying and addressing these disparities first requires improvements in data collection, data quality, and use of data to inform quality improvement initiatives.^27^ Despite national efforts to collect standardized data on race, ethnicity, and primary language, these data are largely incomplete among Medicaid managed care enrollees.^28^ Beyond data collection, states and plans should consider stratifying patient experience measures by race and ethnicity, adopting health equity performance measures,^29,30^ and using data from these measures to develop interventions that address racial and ethnic disparities. For example, California Medicaid managed care plans use a measure of health equity to identify disparities and undertake projects aimed at reducing disparities.^31^ Some Medicaid managed care plans have used enhanced care teams to provide targeted care coordination and care management, whereas others have engaged with community-based organizations to mitigate disparities by connecting enrollees with social services and addressing underlying social determinants of health.^31,32^ Other state strategies to address racial and ethnic health disparities include efforts to improve plans’ cultural competency (for example, identifying preferred languages for communication), enrollee engagement (for example, targeted outreach, development of programs to address identified disparities), and provider engagement (for example, promoting culturally and linguistically diverse provider networks).^5^

More broadly, racial and ethnic disparities in experience of care are driven by structural racism. Therefore, mitigating these disparities requires dismantling the interwoven and mutually reinforcing systems that perpetuate differential access to care and treatment by race and ethnicity in the health care system.^9^ Because these systems are interrelated, initiatives to address them must be comprehensive and cross-sectoral.^9^ For example, in January 2021 CMS issued guidance to state health officials to adopt strategies that address social determinants of health,^33^ building on the existing efforts of Medicaid managed care plans. Although approaches vary, many plans screen for social risk factors, coordinate care with social service providers, and have invested in interventions related to food insecurity or housing instability.^34^ Mitigating racial and ethnic disparities will require efforts from the health care system and cross-sectoral policy reforms that address underlying social inequities and increase financial security and economic opportunity (such as by expanding eligibility and access to other social safety-net programs).^35^

The COVID-19 pandemic and resulting economic crisis have exacerbated existing racial and ethnic inequities in access to care—rising unemployment and subsequent loss of employer-sponsored coverage have disproportionately affected racial and ethnic minority groups, and particularly Black and Hispanic or Latino communities. Because these changes have led to accelerated growth in Medicaid managed care enrollment,^36,37^ Medicaid managed care plans will continue to play a critical role in addressing racial and ethnic disparities throughout and after the pandemic.

## Conclusion

Despite having coverage identical to that of White enrollees, racial and ethnic minority enrollees reported worse experiences of care in Medicaid managed care plans. These results warrant further exploration of state and federal policies, as well as cross-sectoral state- and plan-level strategies, to achieve equity in patient experience throughout the Medicaid managed care program. ■

## Supplementary Material

Supplement

## Figures and Tables

**EXHIBIT 1:** Characteristics of nonelderly Medicaid managed care enrollees and distribution of enrollee race and ethnicity across plans, 2014–18

Enrollee characteristics	White (*n* = 120,887)	Black (*n* = 52,305)	Hispanic or Latino (*n* = 34,336)	Asian American, Native Hawaiian, or other Pacific Islander (*n* = 9,494)
Percent of sample^a^	49.9%	21.6%	14.2%	3.9%
Age, years				
18–44	46.3	49.2	54.5	49.9
45–64	53.7	50.8	45.5	50.2
Female	61.7	65.0	64.7	59.7
More than high school diploma	39.6	35.2	28.7	48.5
Fair or poor self-reported health status	34.8	34.8	32.3	18.6
Survey language: English^b^	99.9	100.0	66.7	99.9
Survey mode: mail	72.9	61.5	64.1	80.0
Assistance reported in completing survey^c^	10.0	9.4	10.8	15.5
Distribution of enrollee race and ethnicity^a,d^	51.3	22.4	17.9	5.7

**source** Authors’ analyses of data from the National Committee on Quality Assurance Adult Medicaid Consumer Assessment of Healthcare Providers and Systems (CAHPS) Health Plan Survey, version 5.0, 2014–18. **notes** Characteristics of these enrollees are shown in appendix exhibit 3 (see note 14 in text). *p* < 0.001 for all comparisons of enrollee characteristics.

a Percentages do not sum to 100 because the main analyses excluded enrollees who were American Indian or Alaska Native, as well as those who were multiracial or reported other race.

b Language in which the respondent completed the survey (options were English or Spanish).

c Indicates that someone had read questions to the respondent, written down answers given, answered questions for the respondent, translated questions into the respondent’s language, or provided other assistance.

d Average percentage of enrollees in each racial and ethnic group across all plans (*n* = 195).

**EXHIBIT 2:** Unadjusted disparities in patient experience of care by race and ethnicity among nonelderly Medicaid managed care enrollees, 2014–18

	Unadjusted disparities (percentage points)		
Experience-of-care measures	Black	Hispanic or Latino	Asian American, Native Hawaiian, or other Pacific Islander
Access to needed care	−2.0	−3.5	−11.6
Access to a personal doctor	−4.5	−6.7	−11.4
Timely access to checkup or routine care	−3.1	−4.8	−15.0
Timely access to specialty care	−3.7	−5.1	−19.3

**source** Authors’ analyses of data from National Committee on Quality Assurance Adult Medicaid Consumer Assessment of Healthcare Providers and Systems (CAHPS) Health Plan Survey, version 5.0, 2014–18. **notes** White enrollees were the reference group; unadjusted disparities are percentage-point differences from the rates reported for White enrollees. Unadjusted rates for White enrollees were 85.7 percent for access to needed care, 84.1 percent for access to a personal doctor, 88.1 percent for timely access to checkup or routine care, and 82.1 percent for timely access to specialty care. *p* < 0.001 for all disparities measured.

**EXHIBIT 3:** Adjusted disparities in patient experience of care by race and ethnicity among nonelderly Medicaid managed care enrollees, 2014–18

	Adjusted disparities (percentage points)		
Experience-of-care measures	Black	Hispanic or Latino	Asian American, Native Hawaiian, or Pacific Islander
Access to needed care			
Overall disparity	−1.5****	−1.6****	−9.8****
Within-plan disparity	−1.2****	−1.1	−9.5****
Between-plan disparity^a^	−0.3	−0.5	−0.3
Access to a personal doctor			
Overall disparity	−4.5****	−3.6****	−9.0****
Within-plan disparity	−4.5****	−3.5****	−8.6****
Between-plan disparity^a^	−0.1	−0.1	−0.3
Timely access to checkup or routine care			
Overall disparity	−2.8****	−3.9****	−13.6****
Within-plan disparity	−2.7****	−3.6****	−13.3****
Between-plan disparity^a^	−0.1	−0.3	−0.2
Timely access to specialty care			
Overall disparity	−3.6****	−3.8****	−17.4****
Within-plan disparity	−3.4****	−3.3****	−16.8****
Between-plan disparity^a^	−0.3	−0.5	−0.6

**source** Authors’ analyses of data from the National Committee on Quality Assurance Adult Medicaid Consumer Assessment of Healthcare Providers and Systems (CAHPS) Health Plan Survey, version 5.0, 2014–18. **notes** White enrollees were the reference group; adjusted disparities are percentage-point differences from the rates reported for White enrollees. “Overall disparity” is the total disparity in patient experience of care across racial and ethnic groups, adjusted for specified characteristics (age, sex, educational attainment, self-reported health status, survey mode [mail versus phone or internet], survey language, receipt of assistance in completing the survey, and state and year fixed effects). “Within-plan disparity” measures differential experiences of care between non-Hispanic White and racial and ethnic minority enrollees within the same plan. “Between-plan disparity” is the difference between overall disparity and within-plan disparity. Between-plan disparities occur when racial and ethnic minorities are disproportionately enrolled in plans that deliver low-quality care for all racial and ethnic groups.

a Consistent with prior work, our main analysis did not test for statistical significance of between-plan disparities. See Goldstein E et al. Racial and ethnic differences in patients’ perceptions of inpatient care using the HCAHPS survey (note 16 in text).

**** *p* < 0.001
