# Supplementary material for: Racial And Ethnic Disparities In Patient Experience Of Care Among Nonelderly Medicaid Managed Care Enrollees
Source: Health Aff (Millwood). Author manuscript; Available in PMC 2023 Apr 6. (PMC10076226; doi:10.1377/hlthaff.2021.01331)
Supplement: Supplement [file NIHMS1786323-supplement-Supplement.pdf]

Nguyen KH, Wilson IB, Wallack AR, Trivedi AN. Racial and ethnic disparities in patient experience of care among nonelderly Medicaid managed care enrollees. Health Aff (Millwood). 2022;41(2).

APPENDIX

Contents

Appendix Exhibit 1. Inclusion and Exclusion Criteria of Study Sample 2

Appendix Exhibit 2. Measure Definitions 3

Appendix Exhibit 3. Characteristics of Nonelderly Medicaid Managed Care Enrollees by Race/Ethnicity, 2014-2018 4

Appendix Exhibit 4. Characteristics of Asian American and Native Hawaiian or Pacific Islander Enrollees, 2014-2018 5

Appendix Exhibit 5. Characteristics of Hispanic/Latino Enrollees by Survey Language, 2014-2018 6

Appendix Exhibit 6. Characteristics of AANHPI Enrollees, by Receipt of Assistance 7

Appendix Exhibit 7. Characteristics of AANHPI, Hispanic/Latino, and Other Race Enrollees, by Receipt of Translation Assistance 8

Appendix Exhibit 8. Unadjusted Rates and Model Estimates 9

Appendix Exhibit 9. Estimates for American Indian and Alaska Native Enrollees, Multiracial Enrollees, and Enrollees Reporting Other Race 10

Appendix Exhibit 10. Estimates for Asian Americans and Native Hawaiians or Pacific Islanders 11

Appendix Exhibit 11. Estimates for AANHPI Enrollees by Receipt of Assistance Completing Survey 12

Appendix Exhibit 12. Estimates for Hispanic/Latino Enrollees by Survey Language 13

Appendix Exhibit 13. Estimates for AANHPI, Hispanic/Latino, and Other Race Enrollees by Receipt of Translation Assistance 14

Appendix Exhibit 14. Multilevel Model Estimates 15

Appendix Exhibit 15. Model Estimates Including Elderly Enrollees and Enrollees Missing Age or Racial/Ethnic Information 16

Appendix Exhibit 16. Model Estimates Excluding American Indian and Alaska Native, Other Race, and Multiracial Enrollees 18

Appendix Exhibit 17. Model Estimates (Top-Box Responses Only) 19

Appendix Exhibit 18. Geographic Variation in Experience of Care by Enrollee Race/Ethnicity 20

Appendix Exhibit 19. White-Black Between-Plan Disparities 21

Appendix Exhibit 20. Relationship Between Proportion of Black Enrollees and White-Black Disparities 22

Appendix Exhibit 21. White-Hispanic/Latino Between-Plan Disparities 23

Appendix Exhibit 22. Relationship Between Proportion of Hispanic/Latino Enrollees and White-Hispanic/Latino Disparities 24

Appendix Exhibit 23. White-AANHPI Between-Plan Disparities 25

Appendix Exhibit 24. Relationship Between Proportion of AANHPI Enrollees and White-AANHPI Disparities 26

Appendix Exhibit 1. Inclusion and Exclusion Criteria of Study Sample

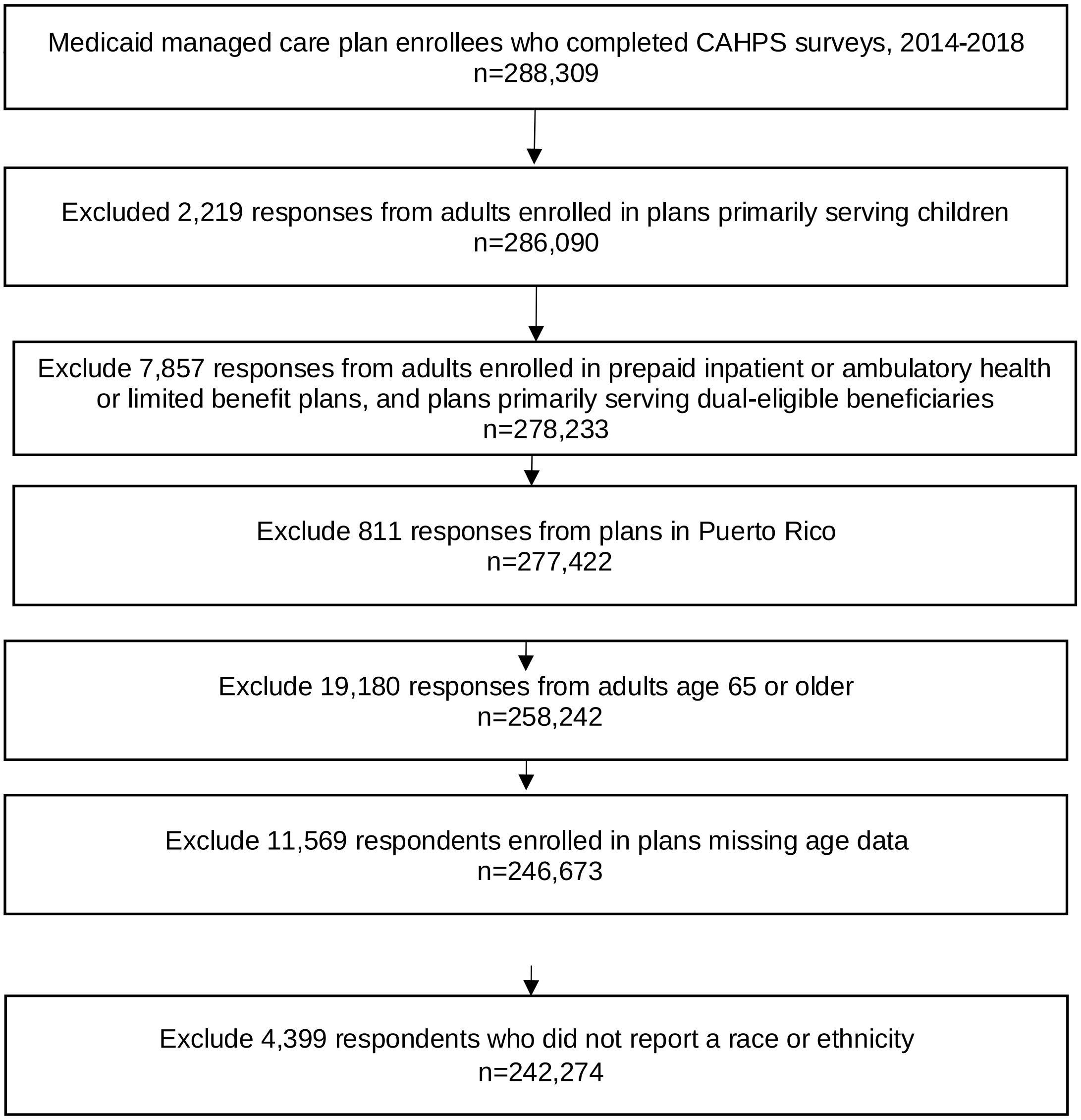

Source: Authors' analyses of National Committee on Quality Assurance (NCQA) Adult Medicaid Consumer Assessment of Healthcare Providers and Systems (CAHPS) Health Plan Survey 5.0, 2014-2018
Note: Counts represent unweighted sample.

Appendix Exhibit 2. Measure Definitions

	CAHPS Question	Responses
**Race/Ethnicity**		
Ethnicity	Are you of Hispanic or Latino origin or descent?	1. Yes, Hispanic or Latino<br>2. No, not Hispanic or Latino
Race	What is your race? Mark one or more.	1. White<br>2. Black or African-American<br>3. Asian<br>4. Native Hawaiian or other Pacific Islander<br>5. American Indian or Alaska Native<br>6. Other
**Outcomes**		
Access to needed care	In the last 6 months, how often was it easy to get the care, tests, or treatment you needed?	1. Never<br>2. Sometimes<br>3. **Usually**<br>4. **Always**
Access to a personal doctor	A personal doctor is the one you would see if you need a check-up, want advice about a health problem, or get sick or hurt. Do you have a personal doctor?	1. **Yes**<br>2. No
Timely access to check up or routine care	In the last 6 months, how often did you get an appointment for a <u>check-up or routine care</u> at a doctor's office or clinic as soon as you needed?	1. Never<br>2. Sometimes<br>3. **Usually**<br>3. **Always**
Timely access to specialty care	Specialists are doctors like surgeons, heart doctors, allergy doctors, skin doctors, and other doctors who specialize in one area of health care. In the last 6 months, how often did you get an appointment to see a specialist as soon as you needed?	1. Never<br>2. Sometimes<br>3. **Usually**<br>4. **Always**

Note. **Bold** indicates response used to define outcome.

Appendix Exhibit 3. Characteristics of Nonelderly Medicaid Managed Care Enrollees by Race/Ethnicity, 2014-2018

	White	Black	Hispanic/ Latino	AANHPI	AIAN	Other Race	Multiracial
**Respondents, n**	120,887	52,305	34,336	9,494	1,559	4,322	19,371
**Proportion of Sample, %**	49.9	21.6	14.2	3.9	0.6	1.8	8.0
**Age, %**							
18-24	11.6	12.8	20.5	15.1	9.4	11.8	13.9
25-34	18.5	18.2	18.8	17.6	13.6	18.7	22.1
35-44	16.3	18.2	15.3	17.1	13.1	20.8	19.1
45-54	22.2	21.7	19.5	19.9	26.8	20.7	20.8
55-64	31.5	29.1	30.3	37.1	28.0	26.1	24.1
**Female, %**	61.7	65.0	64.7	59.7	57.9	58.1	61.7
**Educational Attainment, %**							
8th grade or less	5.0	4.0	14.6	10.6	7.8	5.5	5.5
Some high school, but did not graduate	14.7	20.1	19.0	11.7	19.4	12.0	15.8
High school graduate or GED	39.3	38.9	34.3	26.6	34.3	30.4	33.9
Some college or 2-year degree	29.5	28.2	21.7	25.0	29.2	32.1	31.4
4-year college graduate	6.7	4.6	4.5	15.9	4.7	11.0	6.7
More than 4-year college degree	3.5	2.3	2.5	7.5	2.9	5.6	3.7
**Self-Reported Health Status, %**							
Excellent	9.0	12.5	13.3	13.6	9.4	13.2	11.8
Very Good	22.3	20.5	21.2	28.3	14.7	23.8	21.4
Good	33.1	31.4	32.4	38.3	30.6	31.8	30.0
Fair	24.9	26.9	25.7	15.3	28.6	21.2	24.1
Poor	9.9	7.9	6.6	3.4	15.8	8.5	12.1
**Survey Language - English**	99.9	100.0	66.7	99.9	99.7	99.0	96.0
**Survey Mode**							
Mail	72.9	61.5	64.1	80.0	76.2	75.8	27.2
Telephone	25.7	37.3	34.6	17.4	22.7	23.6	72.1
**Assistance Reported in Completing Survey**	10.0	9.4	10.8	15.5	11.4	10.7	3.3

Source: Authors' analyses of National Committee on Quality Assurance (NCQA) Adult Medicaid Consumer Assessment of Healthcare Providers and Systems (CAHPS) Health Plan Survey 5.0, 2014-2018

Notes. $p<0.001$ using Pearson's chi-square tests for all comparisons. AANHPI refers to Asian American, Native Hawaiian or Pacific Islander. AIAN refers to American Indian or Alaska Native.

Appendix Exhibit 4. Characteristics of Asian American and Native Hawaiian or Pacific Islander Enrollees, 2014-2018

	Asian American	Native Hawaiian or Pacific Islander
**Respondents, n**	8,476	1,018
**Proportion of Sample, %**	3.5	0.4
**Age, %**		
18-24	15.8	8.9
25-34	17.3	22.4
35-44	17.2	16.0
45-54	19.6	23.4
55-64	30.4	29.4
**Female, %**	40.5	37.6
**Educational Attainment, %**		
8th grade or less	11.1	2.5
Some high school, but did not graduate	11.8	10.5
High school graduate or GED	25.8	38.6
Some college or 2-year degree	24.2	36.5
4-year college graduate	16.7	5.0
More than 4-year college degree	7.6	5.8
**Self-Reported Health Status, %**		
Excellent	13.7	12.4
Very Good	28.7	22.2
Good	38.4	36.8
Fair	14.8	21.3
Poor	3.2	6.4
**Survey Language - English**	99.9	100.0
**Survey Mode**		
Mail	80.2	77.7
Telephone	17.1	21.8
**Assistance Reported in Completing Survey**	15.9	10.0

Source: Authors' analyses of National Committee on Quality Assurance (NCQA) Adult Medicaid Consumer Assessment of Healthcare Providers and Systems (CAHPS) Health Plan Survey 5.0, 2014-2018

Appendix Exhibit 5. Characteristics of Hispanic/Latino Enrollees by Survey Language, 2014-2018

	Hispanic-English	Hispanic-Spanish	Hispanic-Indeterminate
**Respondents, n**	23,721	10,345	270
**Proportion of Sample, %**	9.8	4.3	0.1
**Age, %**			
18-24	23.7	13.9	11.5
25-34	22.9	10.5	10.3
35-44	15.7	13.9	30.2
45-54	17.1	24.3	22.8
55-64	20.5	37.4	25.2
**Female, %**	63.9	66.3	69.9
**Educational Attainment, %**			
8th grade or less	7.7	28.7	18.2
Some high school, but did not graduate	17.9	21.4	14.4
High school graduate or GED	37.0	28.9	36.2
Some college or 2-year degree	27.8	9.3	11.9
4-year college graduate	5.2	3.1	4.5
More than 4-year college degree	2.5	2.5	4.0
**Self-Reported Health Status, %**			
Excellent	14.0	11.9	15.5
Very Good	24.6	14.3	14.0
Good	33.0	31.3	29.8
Fair	20.7	35.7	30.2
Poor	6.8	6.1	10.2
**Survey Mode**			
Mail	60.6	71.9	38.1
Telephone	37.6	28.1	61.9
**Assistance Reported in Completing Survey**	10.5	11.5	6.2

Source: Authors' analyses of National Committee on Quality Assurance (NCQA) Adult Medicaid Consumer Assessment of Healthcare Providers and Systems (CAHPS) Health Plan Survey 5.0, 2014-2018

Appendix Exhibit 6. Characteristics of AANHPI Enrollees, by Receipt of Assistance

	AANHPI		Asian American		Native Hawaiian or Pacific Islander	
	**No Assistance**	**Received Assistance**	**No Assistance**	**Received Assistance**	**No Assistance**	**Received Assistance**
**Respondents, n**	8,053	1,441	7,143	1,333	910	108
**Age, %**						
18-24	15.9	11.0	16.4	11.1	8.9	8.6
25-34	18.7	11.8	18.4	11.3	22.3	23.0
35-44	17.6	14.3	17.7	14.4	16.4	12.6
45-54	19.4	22.2	19.1	22.6	24.5	13.5
55-64	28.4	40.7	28.4	40.7	27.9	42.3
**Female, %**	59.2	62.0	58.8	63.2	65.5	34.9
**More than High School Graduate or GED, %**	54.1	17.8	54.4	17.6	50.2	21.0
**Fair/Poor Self-Reported Health Status, %**	17.6	24.3	17.0	23.6	26.3	40.0
**Survey Mode - Mail**	76.7	98.0	76.8	98.0	75.3	99.3
**Assistance Reported in Completing Survey**	-	-	-	-	-	-
Read Questions	-	7.6	-	7.9	-	4.2
Wrote Answers	-	5.3	-	5.5	-	3.2
Answered Questions	-	5.0	-	5.1	-	3.4
Translated Questions	-	11.7	-	12.4	-	1.5
Other Assistance	-	2.5	-	2.5	-	2.5

Source: Authors' analyses of National Committee on Quality Assurance (NCQA) Adult Medicaid Consumer Assessment of Healthcare Providers and Systems (CAHPS) Health Plan Survey 5.0, 2014-2018

Appendix Exhibit 7. Characteristics of AANHPI, Hispanic/Latino, and Other Race Enrollees, by Receipt of Translation Assistance

	AANHPI		Hispanic/Latino		Other Race	
	Did Not Receive Translation Assistance	Received Translation Assistance	Did Not Receive Translation Assistance	Received Translation Assistance	Did Not Receive Translation Assistance	Received Translation Assistance
**Respondents, n**	8,430	1,064	33,412	924	4,186	136
**Respondents, %**	88.8	11.2	97.3	2.7	96.9	3.1
**Age, %**						
18-24	16.9	2.2	20.7	9.7	12.0	5.3
25-34	19.0	7.0	19.1	6.2	19.0	10.4
35-44	17.5	14.2	15.3	13.1	21.0	11.8
45-54	19.1	25.4	19.4	23.3	20.4	30.4
55-64	27.5	51.2	25.5	47.7	27.6	42.2
**Female, %**	58.3	70.0	64.7	65.3	57.9	65.9
**More than High School Graduate or GED, %**	53.4	10.8	29.2	8.8	49.7	16.7
**Fair/Poor Self-Reported Health Status, %**	17.4	28.2	32.2	35.5	29.8	28.7
**Survey Mode - Mail**	77.9	95.9	63.3	98.0	74.0	99.6

Source: Authors' analyses of National Committee on Quality Assurance (NCQA) Adult Medicaid Consumer Assessment of Healthcare Providers and Systems (CAHPS) Health Plan Survey 5.0, 2014-2018

Notes: "Received Translation Assistance" includes enrollees who indicated that someone helped them complete the CAHPS survey and "translated the questions into my language."

Appendix Exhibit 8. Unadjusted Rates and Model Estimates

Access to Needed Care	Unadjusted Rates, %	Unadjusted	Model 1	Model 2	Model 3
White	85.7	Ref	Ref	Ref	Ref
Black	83.7****	-2.0****	-2.1****	-1.5****	-1.2****
Hispanic/Latino	82.2****	-3.5****	-4.8****	-1.6****	-1.1**
Asian, Native Hawaiian, or Pacific Islander	75.5****	-11.6****	-12.9****	-9.8****	-9.5****
American Indian or Alaska Native	77.2****	-8.5****	-8.1****	-7.2****	-7.2****
Other Race	75.5****	-10.2****	-10.4****	-8.6****	-8.1****
Multiracial	78.7****	-7.0****	-5.4****	-4.4****	-4.2****
**Access to a Personal Doctor**	**Unadjusted Rates, %**	**Unadjusted**	**Model 1**	**Model 2**	**Model 3**
White	84.1	Ref	Ref	Ref	Ref
Black	79.6****	-4.5****	-4.3****	-4.5****	-4.5****
Hispanic/Latino	77.4****	-6.7****	-4.9****	-3.6****	-3.5****
Asian, Native Hawaiian, or Pacific Islander	72.6****	-11.4****	-10.3****	-9.0****	-8.6****
American Indian or Alaska Native	75.7***	-8.4****	-9.7****	-7.9****	-7.9****
Other Race	79.5****	-4.5****	-3.8****	-3.8****	-3.7****
Multiracial	80.4****	-3.7****	-2.6****	-2.5****	-2.4****
**Access to Checkup or Routine Care**	**Unadjusted Rates, %**	**Unadjusted**	**Model 1**	**Model 2**	**Model 3**
White	88.1	Ref	Ref	Ref	Ref
Black	85.0****	-3.1****	-2.8****	-2.8****	-2.7****
Hispanic/Latino	83.2****	-4.8****	-5.4****	-3.9****	-3.6****
Asian, Native Hawaiian, or Pacific Islander	73.1****	-15.0****	-15.3****	-13.6****	-13.3****
American Indian or Alaska Native	84.5****	-2.3	-2.5***	-2.2	-2.2
Other Race	79.9****	-8.2****	-8.3****	-7.3****	-7.0****
Multiracial	82.0****	-6.1****	-4.7****	-4.2****	-4.1****
**Access to a Specialty Care Appointment**	**Unadjusted Rates, %**	**Unadjusted**	**Model 1**	**Model 2**	**Model 3**
White	82.1	Ref	Ref	Ref	Ref
Black	78.4****	-3.7****	-3.8****	-3.6****	-3.4****
Hispanic/Latino	77.1****	-5.1****	-6.6****	-3.8****	-3.3****
Asian, Native Hawaiian, or Pacific Islander	62.8****	-19.3****	-20.2****	-17.4****	-16.8****
American Indian or Alaska Native	75.2****	-6.9***	-7.4****	-6.5**	-6.2**
Other Race	70.6****	-11.5****	-11.7****	-10.0****	-9.6****
Multiracial	74.1****	-8.0****	-6.0****	-5.2****	-5.1****

Source: Authors' analyses of National Committee on Quality Assurance (NCQA) Adult Medicaid Consumer Assessment of Healthcare Providers and Systems (CAHPS) Health Plan Survey 5.0, 2014-2018

Note. **** p<0.001, *** p<0.01, ** p<0.05, * p<0.1. **Model 1** adjusts for age, gender, educational attainment, self-reported health status, survey model (mail vs. phone or internet), survey language, receipt of assistance in completing the survey, and year fixed effects. **Model 2** adds state fixed effects. **Model 3** adds plan fixed effects. All models are linear probability regression models.

Appendix Exhibit 9. Estimates for American Indian and Alaska Native Enrollees, Multiracial Enrollees, and Enrollees Reporting Other Race

	American Indian and Alaska Natives	Other Race	Multiracial
**Access to Needed Care**			
Unadjusted Rate, %	77.2****	75.5****	78.7****
Unadjusted Disparity	-8.5****	-10.2****	-7.0****
Overall Disparity	-7.2****	-8.6****	-4.4****
Within-Plan Disparity	-7.2****	-8.1****	-4.2****
Between-Plan Disparity	0.0	-0.4	-0.2
**Access to a Personal Doctor**			
Unadjusted Rate, %	75.7**	79.5****	80.4****
Unadjusted Disparity	-8.4****	-4.5****	-3.7****
Overall Disparity	-7.9****	-3.8****	-2.5****
Within-Plan Disparity	-7.9****	-3.7****	-2.4****
Between-Plan Disparity	-0.1	-0.2	-0.1
**Timely Access to Check-Up or Routine Care**			
Unadjusted Rate, %	84.5****	79.9****	82.0****
Unadjusted Disparity	-2.3	-8.2****	-6.1****
Overall Disparity	-2.2****	-7.3****	-4.2****
Within-Plan Disparity	-2.2****	-7.0****	-4.1****
Between-Plan Disparity	-0.1	-0.3	-0.1
**Timely Access to Specialty Care**			
Unadjusted Rate, %	75.2****	70.6****	74.1****
Unadjusted Disparity	-6.9**	-11.5****	-8.0****
Overall Disparity	-6.5	-10.0****	-5.2****
Within-Plan Disparity	-6.2	-9.6****	-5.1****
Between-Plan Disparity	-0.3	-0.4	-0.1

Source: Authors' analyses of National Committee on Quality Assurance (NCQA) Adult Medicaid Consumer Assessment of Healthcare Providers and Systems (CAHPS) Health Plan Survey 5.0, 2014-2018

Note. **** p<0.001, *** p<0.01, ** p<0.05, * p<0.1. "Overall Disparity" adjusts for age, gender, educational attainment, self-reported health status, survey model (mail vs. phone or internet), survey language, receipt of assistance in completing the survey, and state and year fixed effects. "Within-Plan Disparity" adds a plan fixed effect. "Between-Plan" disparity is the difference between "Overall Disparity" and "Within-Plan Disparity"

Appendix Exhibit 10. Estimates for Asian Americans and Native Hawaiians or Pacific Islanders

	Asian Americans	Native Hawaiians or Pacific Islanders
**Access to Needed Care**		
Unadjusted Rate, %	73.5*****	82.7
Unadjusted Disparity	-12.2****	-3.0
**Access to a Personal Doctor**		
Unadjusted Rate, %	72.6****	72.7****
Unadjusted Disparity	-11.4****	-11.4****
**Timely Access to Check-Up or Routine Care**		
Unadjusted Rate, %	72.5****	81.6**
Unadjusted Disparity	-15.6****	-6.4**
**Timely Access to Specialty Care**		
Unadjusted Rate, %	61.5****	78.9
Unadjusted Disparity	-20.6****	-3.2

Source: Authors' analyses of National Committee on Quality Assurance (NCQA) Adult Medicaid Consumer Assessment of Healthcare Providers and Systems (CAHPS) Health Plan Survey 5.0, 2014-2018

Note. **** p<0.001, *** p<0.01, ** p<0.05, * p<0.1. Only unadjusted estimates are presented because of small sample size for Native Hawaiian and Pacific Islander respondents.

Appendix Exhibit 11. Estimates for AANHPI Enrollees by Receipt of Assistance Completing Survey

	Did Not Receive Assistance	Received Assistance
**Access to Needed Care**		
Unadjusted Rate, %	74.2****	73.8****
Unadjusted Disparity	-11.5****	-11.9****
Overall Disparity	-9.7****	-10.8****
Within-Plan Disparity	-9.3****	-10.5****
Between-Plan Disparity	-0.4	-0.3
**Access to a Personal Doctor**		
Unadjusted Rate, %	71.9****	76.5****
Unadjusted Disparity	-12.1****	-7.6****
Overall Disparity	-8.6****	-11.0****
Within-Plan Disparity	-8.3****	-10.4****
Between-Plan Disparity	-0.3	-0.6
**Timely Access to Check-Up or Routine Care**		
Unadjusted Rate, %	72.9****	73.8****
Unadjusted Disparity	-15.1****	-14.2****
Overall Disparity	-14.9****	-17.0****
Within-Plan Disparity	-13.1****	-14.6****
Between-Plan Disparity	-1.8	-2.4
**Timely Access to Specialty Care**		
Unadjusted Rate, %	61.5****	68.6****
Unadjusted Disparity	-20.6****	-13.5****
Overall Disparity	-18.2****	-14.0****
Within-Plan Disparity	-17.6****	-13.4***
Between-Plan Disparity	-0.6	-0.6

Source: Authors' analyses of National Committee on Quality Assurance (NCQA) Adult Medicaid Consumer Assessment of Healthcare Providers and Systems (CAHPS) Health Plan Survey 5.0, 2014-2018

Note. **** p<0.001, *** p<0.01, ** p<0.05, * p<0.1. AANHPI refers to Asian American, Native Hawaiian or Pacific Islander. "Assistance Completing Survey" included: having questions read, writing down answers given, answering the questions for the respondent, translating questions into the respondent's language, or other assistance. Reference group is non-Hispanic White Medicaid managed care enrollees. "Overall Disparity" adjusts for age, gender, educational attainment, self-reported health status, survey model (mail vs. phone or internet), survey language, receipt of assistance in completing the survey, and state and year fixed effects. "Within-Plan Disparity" adds a plan fixed effect. "Between-Plan" disparity is the difference between "Overall Disparity" and "Within-Plan Disparity." We tested the interaction between being AANHPI and receiving assistance to assess if there were statistical differences between outcomes for those who received assistance vs. those who did not, and no coefficients were statistically significant.

Appendix Exhibit 12. Estimates for Hispanic/Latino Enrollees by Survey Language

	Hispanic/Latino-English	Hispanic/Latino-Spanish	Hispanic/Latino-Indeterminate
**Access to Needed Care**			
Unadjusted Rate, %	80.8****	85.1	80.0**
Unadjusted Disparity	-4.9****	-0.5	-5.7**
Overall Disparity	-1.6****	-1.1	-
Within-Plan Disparity	-1.1*	-0.7	-
Between-Plan Disparity	-0.5	-0.4	-
**Access to a Personal Doctor**			
Unadjusted Rate, %	76.8****	78.5****	87.7
Unadjusted Disparity	-7.3****	-5.6****	3.7
Overall Disparity	-3.5****	-4.9****	-
Within-Plan Disparity	-3.5****	-5.1**	-
Between-Plan Disparity	0.0	0.2	-
**Timely Access to Check-Up or Routine Care**			
Unadjusted Rate, %	81.8****	86.1**	88.1
Unadjusted Disparity	-6.2****	-2.0**	-0.6
Overall Disparity	-3.9****	-3.2****	-
Within-Plan Disparity	-3.6****	-3.1**	-
Between-Plan Disparity	-0.3	-0.1	-
**Timely Access to Specialty Care**			
Unadjusted Rate, %	74.8****	81.5	73.4
Unadjusted Disparity	-7.3****	-0.6	-8.7
Overall Disparity	-3.7****	-4.9*	-
Within-Plan Disparity	-3.3****	-4.6	-
Between-Plan Disparity	-0.4	-0.3	-

Source: Authors' analyses of National Committee on Quality Assurance (NCQA) Adult Medicaid Consumer Assessment of Healthcare Providers and Systems (CAHPS) Health Plan Survey 5.0, 2014-2018

Note. **** p<0.001, *** p<0.01, ** p<0.05, * p<0.1. Language item on the survey identified the language in which the enrollee completed the survey. "Overall Disparity" adjusts for age, gender, educational attainment, self-reported health status, survey model (mail vs. phone or internet), survey language, receipt of assistance in completing the survey, and state and year fixed effects. "Within-Plan Disparity" adds a plan fixed effect. "Between-Plan" disparity is the difference between "Overall Disparity" and "Within-Plan Disparity." Only unadjusted estimates are presented for Hispanic/Latino enrollees with indeterminate survey language because of small sample size.

Appendix Exhibit 13. Estimates for AANHPI, Hispanic/Latino, and Other Race Enrollees by Receipt of Translation Assistance

	Received Translation Assistance	Did Not Receive Translation Assistance	p-value
**Asian American, Native Hawaiian, or Pacific Islander, %**	**11.7**	**88.3**	**-**
Access to Needed Care	72.2	74.4	0.49
Access to a Personal Doctor	81.4	71.5	0.01**
Timely Access to Check-Up or Routine Care	68.8	73.6	0.16
Timely Access to Specialty Care	61.6	63.0	0.31
**Hispanic/Latino, %**	**2.4**	**97.6**	-
Access to Needed Care	80.1	82.2	0.42
Access to a Personal Doctor	84.7	77.2	0.02**
Timely Access to Check-Up or Routine Care	78.9	83.3	0.06*
Timely Access to Specialty Care	75.8	70.7	0.16
**Other Race, %**	**3.0**	**97.0**	-
Access to Needed Care	75.7	75.5	0.47
Access to a Personal Doctor	81.1	79.5	0.96
Timely Access to Check-Up or Routine Care	71.8	80.1	0.85
Timely Access to Specialty Care	70.7	70.6	0.39

Source: Authors' analyses of National Committee on Quality Assurance (NCQA) Adult Medicaid Consumer Assessment of Healthcare Providers and Systems (CAHPS) Health Plan Survey 5.0, 2014-2018

Notes. *p<0.1, **p<0.05, ***p<0.01, ****p<0.001. "Received Translation Assistance" includes enrollees who indicated that someone helped them complete the CAHPS survey and "translated the questions into my language." "Model adjusts for race/ethnicity, receipt of translation assistance, and its interaction. P-value reflects the statistical significance of race/ethnicity x assistance. Only unadjusted estimates are presented because of small sample size.

Appendix Exhibit 14. Multilevel Model Estimates

Access to Needed Care	Unadjusted	Model 1	Model 2	Model 3
White	Ref	Ref	Ref	Ref
Black	-2.6****	-2.5****	-1.6****	-1.3****
Hispanic/Latino	-3.6****	-4.6****	-2.7****	-2.4****
Asian, Native Hawaiian, or Pacific Islander	-11.3****	-12.4****	-11.4****	-11.2****
American Indian or Alaska Native	-7.5****	-7.0****	-6.7****	-6.7****
Other Race	-9.4****	-9.6****	-8.6****	-8.3****
Multiracial	-6.8****	-5.1****	-4.6****	-4.5****
**Access to a Personal Doctor**	**Unadjusted**	**Model 1**	**Model 2**	**Model 3**
White	Ref	Ref	Ref	Ref
Black	-4.4****	-4.2****	-4.4****	-4.3****
Hispanic/Latino	-6.1****	-4.9****	-3.3****	-3.4****
Asian, Native Hawaiian, or Pacific Islander	-11.6****	-10.3****	-8.9****	-9.0****
American Indian or Alaska Native	-10.4****	-11.4****	-9.6****	-9.4****
Other Race	-5.1****	-4.6****	-4.5****	-4.4****
Multiracial	-4.3****	-3.1****	-2.7****	-2.6****
**Access to Checkup or Routine Care**	**Unadjusted**	**Model 1**	**Model 2**	**Model 3**
White	Ref	Ref	Ref	Ref
Black	-3.6****	-3.3****	-3.1****	-3.0****
Hispanic/Latino	-5.0****	-5.6****	-4.7****	-4.5****
Asian, Native Hawaiian, or Pacific Islander	-14.0****	-14.1****	-13.7****	-13.6****
American Indian or Alaska Native	-3.5****	-3.5****	-3.5****	-3.5****
Other Race	-7.5****	-7.6****	-7.1****	-7.0****
Multiracial	-5.9****	-4.4****	-4.3****	-4.3****
**Access to a Specialty Care Appointment**	**Unadjusted**	**Model 1**	**Model 2**	**Model 3**
White	Ref	Ref	Ref	Ref
Black	-4.1****	-4.1****	-3.5****	-3.4****
Hispanic/Latino	-4.7****	-6.4****	-4.7****	-4.6****
Asian, Native Hawaiian, or Pacific Islander	-18.8****	-19.2****	-18.3****	-18.1****
American Indian or Alaska Native	-5.0**	-5.5****	-4.8****	-4.8****
Other Race	-9.0****	-9.1****	-8.1****	-8.0****
Multiracial	-7.0****	-4.9****	-4.4****	-4.4****

Source: Authors' analyses of National Committee on Quality Assurance (NCQA) Adult Medicaid Consumer Assessment of Healthcare Providers and Systems (CAHPS) Health Plan Survey 5.0, 2014-2018

Note. **** p<0.001, *** p<0.01, ** p<0.05, * p<0.1. **Model 1** adjusts for age, gender, educational attainment, self-reported health status, survey model (mail vs. phone or internet), survey language, receipt of assistance in completing the survey, and year fixed effects. **Model 2** adds state fixed effects. **Model 3** adds a plan random effect.

Appendix Exhibit 15. Model Estimates Including Elderly Enrollees and Enrollees Missing Age or Racial/Ethnic Information

**Access to Needed Care**	**Unadjusted Average**	**Unadjusted Difference**	**Model 1**	**Model 2**	**Model 3**
White	86.0	Ref	Ref	Ref	Ref
Missing Race/Ethnicity	77.2****	-8.8****	-7.1****	-6.4****	-6.1****
Black	83.9****	-2.1****	-2.2****	-1.6****	-1.3***
Hispanic/Latino	82.9****	-3.2****	-4.8****	-1.8***	-1.4**
Asian, Native Hawaiian, or Pacific Islander (AANHPI)	75.2****	-10.8****	-13.0****	-9.8****	-9.6***
American Indian or Alaska Native (AIAN)	77.7****	-8.3****	-8.1****	-7.3****	-7.3***
Other Race	76.4****	-9.6****	-9.9****	-8.1****	-7.7***
Multiracial	78.9****	-7.1****	-5.4****	-4.5****	-4.4***
**Access to a Personal Doctor**	**Unadjusted Average**	**Unadjusted**	**Model 1**	**Model 2**	**Model 3**
White	84.5	Ref	Ref	Ref	Ref
Missing Race/Ethnicity	76.7****	-7.7****	-6.5****	-6.4****	-6.3****
Black	80.1****	-4.4****	-4.0****	-4.3****	-4.2****
Hispanic/Latino	78.8****	-5.7****	-4.6****	-3.4****	-3.3****
AANHPI	74.4****	-10.0****	-10.0****	-8.8****	-8.6****
AIAN	75.8****	-8.6****	-10.1****	-8.2****	-8.2 ****
Other Race	80.0****	-4.8****	-4.2****	-4.2****	-4.1 ****
Multiracial	80.8****	-3.7****	-2.6****	-2.5****	-2.4****
**Access to Check-up or Routine Care**	**Unadjusted Average**	**Unadjusted**	**Model 1**	**Model 2**	**Model 3**
White	88.2	Ref	Ref	Ref	Ref
Missing Race/Ethnicity	86.6****	-1.6****	-3.9****	-3.5****	-3.4 ****
Black	85.1****	-3.1****	-2.8****	-2.8****	-2.6 ****
Hispanic/Latino	83.6****	-4.5****	-5.5****	-3.9****	-3.6 ****
AANHPI	73.3****	-14.8****	-15.4****	-13.6****	-13.3 ****
AIAN	85.9	-2.3	-2.6***	-2.3	-2.2
Other Race	80.0****	-8.3****	-8.4****	-7.3****	-7.0 ***
Multiracial	82.1****	-6.1****	-4.7****	-4.2****	-4.1 ***
**Access to a Specialty Care Appointment**	**Unadjusted Average**	**Unadjusted**	**Model 1**	**Model 2**	**Model 3**
White	82.3	Ref	Ref	Ref	Ref
Missing Race/Ethnicity	72.4****	-9.9***	-8.8****	-8.0****	-7.8 ****
Black	78.7****	-3.6***	-3.7****	-3.5****	-3.3****
Hispanic/Latino	78.4****	-4.0***	-6.7****	-3.9****	-3.5***
AANHPI	63.1****	-19.2***	-20.8****	-17.7***	-17.1****
AIAN	75.3**	-7.0**	-7.6****	-6.6**	-6.3*
Other Race	70.1****	-12.2***	-12.4****	-10.7****	-10.3****
Multiracial	74.4****	-8.0***	-6.1****	-5.3****	-5.2****

Source: Authors' analyses of National Committee on Quality Assurance (NCQA) Adult Medicaid Consumer Assessment of Healthcare Providers and Systems (CAHPS) Health Plan Survey 5.0, 2014-2018

Note. **** p<0.001, *** p<0.01, ** p<0.05, * p<0.1. Sample includes enrollees who were elderly (age 65 or older) or who were missing data on age, race, or ethnicity. **Model 1**

adjusts for age, gender, educational attainment, self-reported health status, survey model (mail vs. phone or internet), survey language, receipt of assistance in completing the survey, and year fixed effects. **Model 2** adds state fixed effects. **Model 3** adds plan fixed effects. All models are linear probability regression models.

Appendix Exhibit 16. Model Estimates Excluding American Indian and Alaska Native, Other Race, and Multiracial Enrollees

	White	Black	Hispanic/ Latino	Asian, Native Hawaiian, or Pacific Islander
**Access to Needed Care**				
Overall Disparity	Ref	-1.5****	-1.5****	-9.8****
Within-Plan Disparity	Ref	-1.3****	-1.0	-9.5****
Between-Plan Disparity	Ref	-0.2	-0.5	-0.3
**Access to a Personal Doctor**				
Overall Disparity	Ref	-4.5****	-3.6****	-8.9****
Within-Plan Disparity	Ref	-4.5****	-3.5****	-8.6****
Between-Plan Disparity	Ref	0.0	-0.1	-0.3
**Timely Access to Checkup or Routine Care**				
Overall Disparity	Ref	-2.9****	-3.9****	-13.6****
Within-Plan Disparity	Ref	-2.7****	-3.6****	-13.4****
Between-Plan Disparity	Ref	-0.1	-0.3	-0.2
**Timely Access to Specialty Care**				
Overall Disparity	Ref	-3.6****	-4.0****	-17.8****
Within-Plan Disparity	Ref	-3.4****	-3.6****	-17.1****
Between-Plan Disparity	Ref	-0.3	-0.4	-0.7

Source: Authors' analyses of National Committee on Quality Assurance (NCQA) Adult Medicaid Consumer Assessment of Healthcare Providers and Systems (CAHPS) Health Plan Survey 5.0, 2014-2018

Note. **** p<0.001, *** p<0.01, ** p<0.05, * p<0.1. "Overall Disparity" adjusts for age, gender, educational attainment, self-reported health status, survey model (mail vs. phone or internet), survey language, receipt of assistance in completing the survey, and state and year fixed effects. "Within-Plan Disparity" adds a plan fixed effect. "Between-Plan" disparity is the difference between "Overall Disparity" and "Within-Plan Disparity"

Appendix Exhibit 17. Model Estimates (Top-Box Responses Only)

**Access to Needed Care**	**Unadjusted Rates, %**	**Unadjusted**	**Model 1**	**Model 2**	**Model 3**
White	56.4	Ref	Ref	Ref	Ref
Black	62.0***	5.6***	4.1***	4.2***	4.4***
Hispanic/Latino	52.7***	-3.7***	-4.8***	-0.5	-0.1
Asian, Native Hawaiian, or Pacific Islander	40.2***	-16.2***	-17.3***	-12.7***	-12.2***
American Indian or Alaska Native	51.4**	-5.0**	-5.1***	-3.7	-3.7
Other Race	44.8***	-11.6***	-11.7***	-9.1***	-8.7***
Multiracial	53.3***	-3.1***	-4.5***	-3.2***	-3.0***
**Access to Check-up or Routine Care**	**Unadjusted Rates, %**	**Unadjusted**	**Model 1**	**Model 2**	**Model 3**
White	58.7	Ref	Ref	Ref	Ref
Black	59.5	0.8	0.4	-0.1	0.2
Hispanic/Latino	50.0***	-8.6***	-9.5***	-5.9***	-5.5***
Asian, Native Hawaiian, or Pacific Islander	31.8***	-26.9***	-26.7***	-22.2***	-22.0***
American Indian or Alaska Native	56.1	-2.6	-3.4**	-2.1	-2.0
Other Race	45.4***	-13.2***	-13.0***	-10.7***	-10.4***
Multiracial	53.7***	-5.0***	-4.6***	-3.7***	-3.5***
**Access to a Specialty Care Appointment**	**Unadjusted Rates, %**	**Unadjusted**	**Model 1**	**Model 2**	**Model 3**
White	55.4	Ref	Ref	Ref	Ref
Black	55.9	0.5	-0.4	-0.4	-0.1
Hispanic/Latino	49.4***	-6.0***	-7.5***	-4.2***	-3.8***
Asian, Native Hawaiian, or Pacific Islander	33.0***	-22.4***	-22.9***	-18.9***	-18.8***
American Indian or Alaska Native	50.5	-4.8	-4.8**	-3.7	-3.5
Other Race	43.3***	-12.0***	-12.3***	-10.1***	-9.7***
Multiracial	51.0***	-4.3***	-4.5***	-3.6***	-3.5***

Source: Authors' analyses of National Committee on Quality Assurance (NCQA) Adult Medicaid Consumer Assessment of Healthcare Providers and Systems (CAHPS) Health Plan Survey 5.0, 2014-2018

Note. **** p<0.001, *** p<0.01, ** p<0.05, * p<0.1. "Access to Needed Care" defined as responding "Always" to survey item that asked "In the last 6 months, how often was it easy to get the care, tests, or treatment you needed?" "Access to Check-up or Routine Care defined as responding "Always" to survey item that "In the last 6 months, how often did you get an appointment for a check-up or routine care at a doctor's office or clinic as soon as you needed?" "Access to Specialty Care Appointment" defined as responding "Always" to survey item that "Specialists are doctors like surgeons, heart doctors, allergy doctors, skin doctors, and other doctors who specialize in one area of health care. In the last 6 months, how often did you get an appointment to see a specialist as soon as you needed?" Estimates are not included for "Access to Personal Doctor" because it is a binary outcome, while these three outcomes are on Likert scales.

Appendix Exhibit 18. Geographic Variation in Experience of Care by Enrollee Race/Ethnicity

	Northeast (Ref)	South	Midwest	West
**Access to Needed Care, %**				
White (Ref)	82.4	83.6	83.3	75.2
Black	74.5	79.7	78.9	72.5
Hispanic/Latino	76.3	77.3	75.4	74.2****
AANHPI	59.4	61.9	56.8	63.7*
AIAN	83.9	77.2	82.4	65.5
Other	70.1	73.0	73.9	65.9
Multiracial	75.5	78.0	78.6	71.2**
**Access to a Personal Doctor, %**				
White (Ref)	88.9	82.8	85.6	77.7
Black	84.1	78.7	80.7	76.5**
Hispanic/Latino	86.5	80.0	82.7	74.4
AANHPI	82.9	72.3**	73.1***	70.6
AIAN	85.3	78.3	80.9	65.0**
Other	85.4	79.8	81.2	75.7
Multiracial	88.5	80.0**	83.4	76.2
**Timely Access to a Checkup or Routine Care, %**				
White (Ref)	88.2	89.1	88.3	83.6
Black	82.8	85.8*	85.7**	81.5*
Hispanic/Latino	82.3	83.4	81.6	82.3****
AANHPI	70.8	75.0*	73.7	72.3***
AIAN	89.0	87.7	87.0	82.5
Other	79.8	82.2	80.7	76.7
Multiracial	81.2	84.5	83.9	82.1****
**Timely Access to Specialty Care, %**				
White (Ref)	82.4	83.6	83.3	75.2
Black	74.5	79.7****	78.9***	72.5****
Hispanic/Latino	76.3	77.3	75.4	74.2***
AANHPI	59.4	61.9	56.8	63.7***
AIAN	83.9	77.2	82.4	65.5
Other	70.1	73.0	73.9	65.9
Multiracial	75.5	78.0	78.6	71.2

Source: Authors' analyses of National Committee on Quality Assurance (NCQA) Adult Medicaid Consumer Assessment of Healthcare Providers and Systems (CAHPS) Health Plan Survey 5.0, 2014-2018

Notes. *p<0.1, **p<0.05, ***p<0.01, ****p<0.001. Estimates in exhibit are predicted values obtained using "MARGINS" command in Stata, where models include an indicator for race/ethnicity, Census region, and their interaction (race/ethnicity x Census region) with Northeast as the reference region. Models also adjust for age, gender, educational attainment, self-reported health status, survey model (mail vs. phone or internet), survey language, receipt of assistance in completing the survey. P-value tests statistical significance of race/ethnicity x Census region interaction.

Appendix Exhibit 19. White-Black Between-Plan Disparities

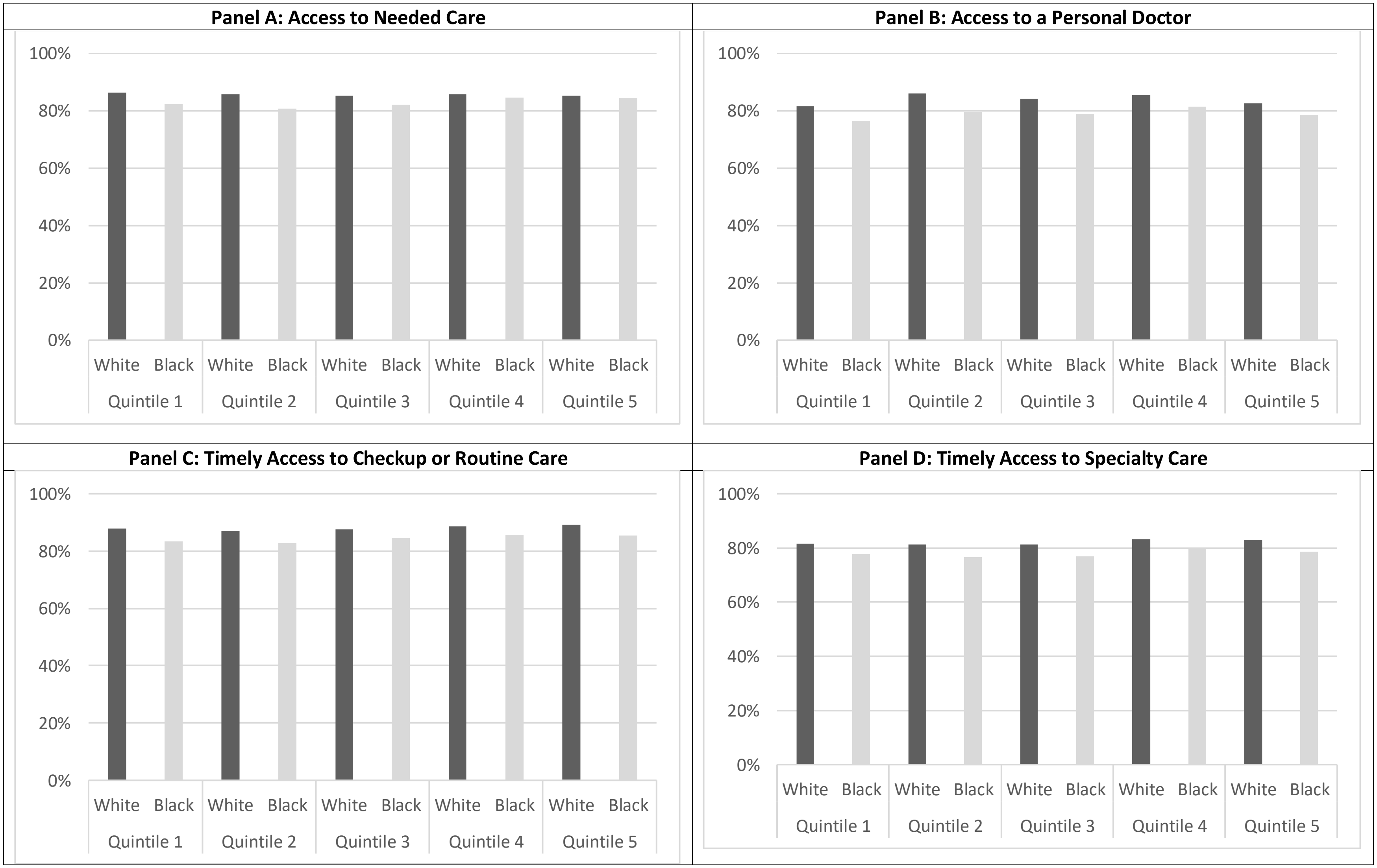

Source: Authors' analyses of National Committee on Quality Assurance (NCQA) Adult Medicaid Consumer Assessment of Healthcare Providers and Systems (CAHPS) Health Plan Survey 5.0, 2014-2018.

Notes. Quintiles reflect plan-level proportion of enrollees who are Black. Quintile 1 represents the 20% of plans with the lowest proportion of Black enrollees and Quintile 5 represents the 20% of plans with the highest proportion of Black enrollees.

Appendix Exhibit 20. Relationship Between Proportion of Black Enrollees and White-Black Disparities

Panel A: Access to Needed Care

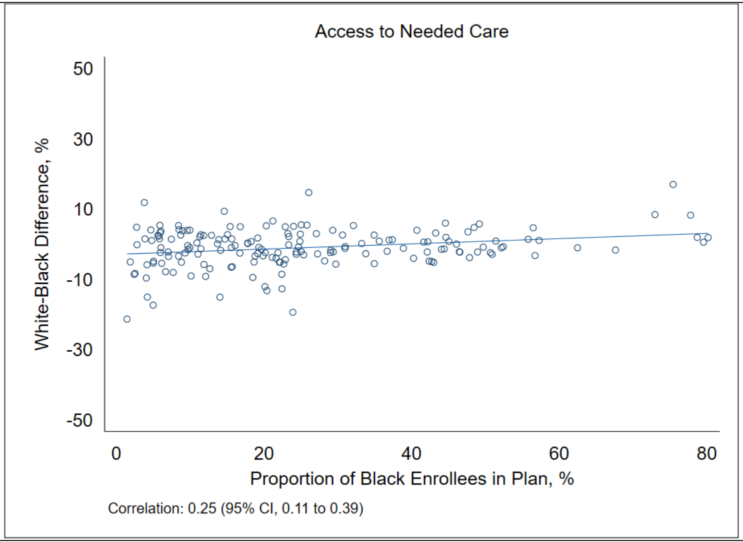

Panel B: Access to a Personal Doctor

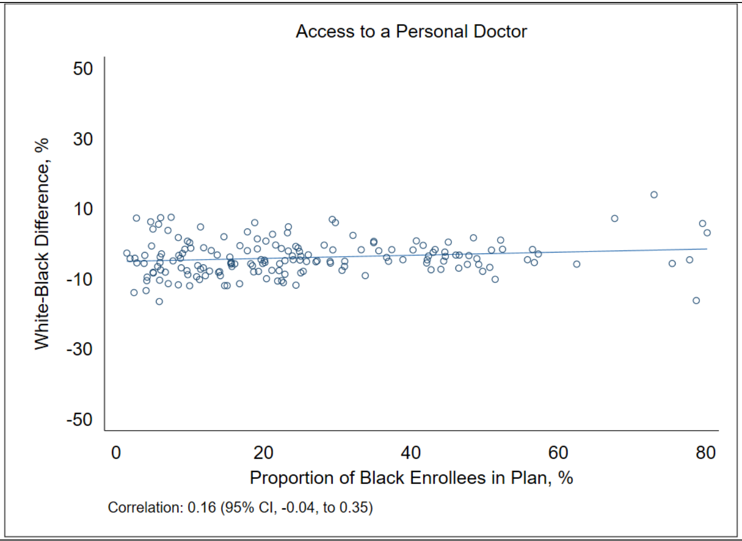

Panel C: Timely Access to Checkup or Routine Care

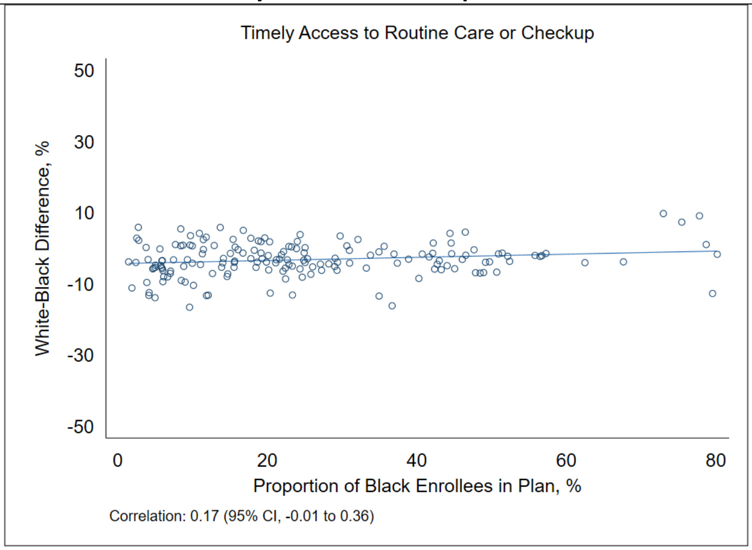

Panel D: Timely Access to Specialty Care

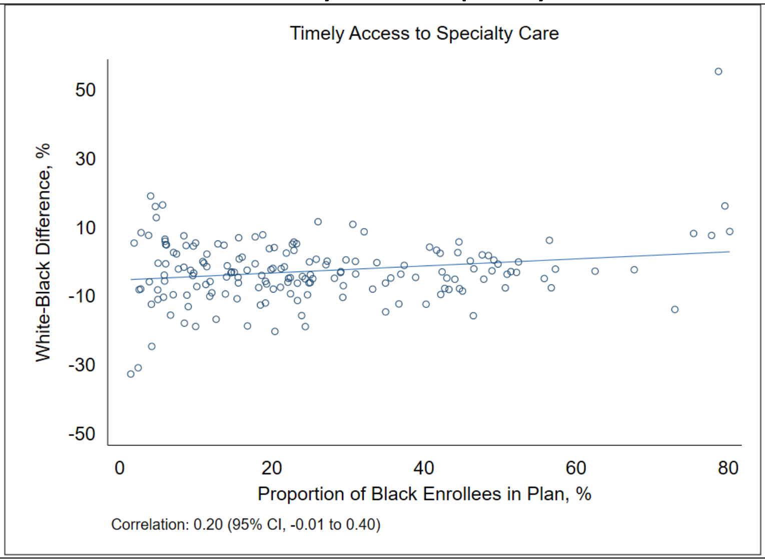

Source: Authors' analyses of National Committee on Quality Assurance (NCQA) Adult Medicaid Consumer Assessment of Healthcare Providers and Systems (CAHPS) Health Plan Survey 5.0, 2014-2018

Note. Analyses are at the plan level. Correlation calculated using Pearson's correlation coefficient and 95% CI calculated by bootstrapping standard errors with 10,000 replications.

Appendix Exhibit 21. White-Hispanic/Latino Between-Plan Disparities

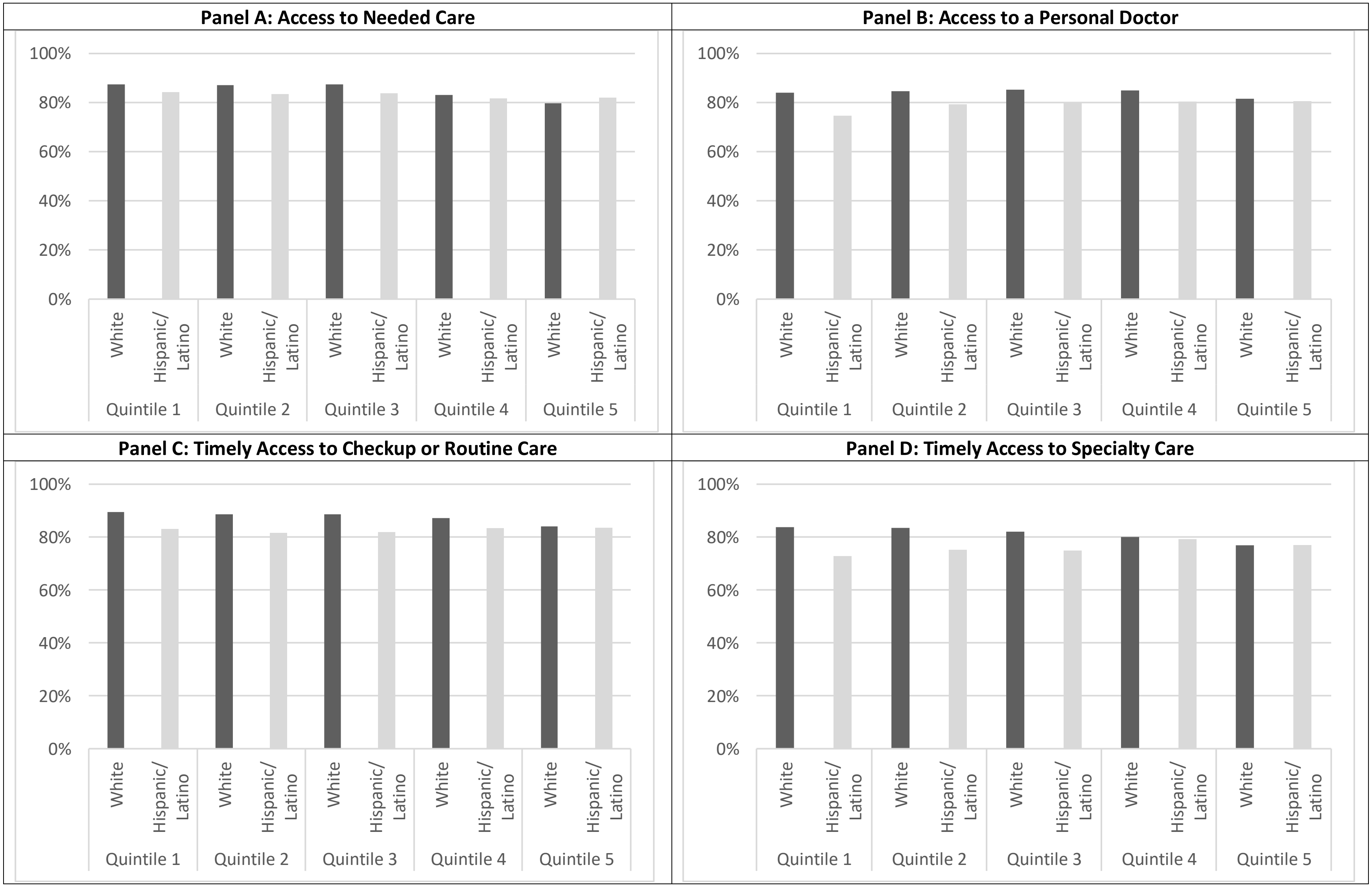

Source: Authors' analyses of National Committee on Quality Assurance (NCQA) Adult Medicaid Consumer Assessment of Healthcare Providers and Systems (CAHPS) Health Plan Survey 5.0, 2014-2018.

Notes: Quintiles reflect plan-level proportion of enrollees who are Hispanic/Latino. Quintile 1 represents the 20% of plans with the lowest proportion of Hispanic/Latino enrollees and Quintile 5 represents the 20% of plans with the highest proportion of Hispanic/Latino enrollees.

Appendix Exhibit 22. Relationship Between Proportion of Hispanic/Latino Enrollees and White-Hispanic/Latino Disparities

Panel A: Access to Needed Care

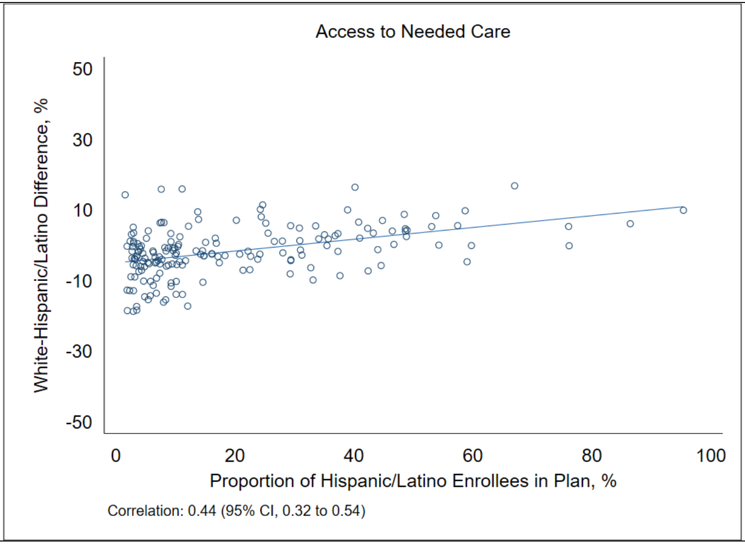

Panel B: Access to a Personal Doctor

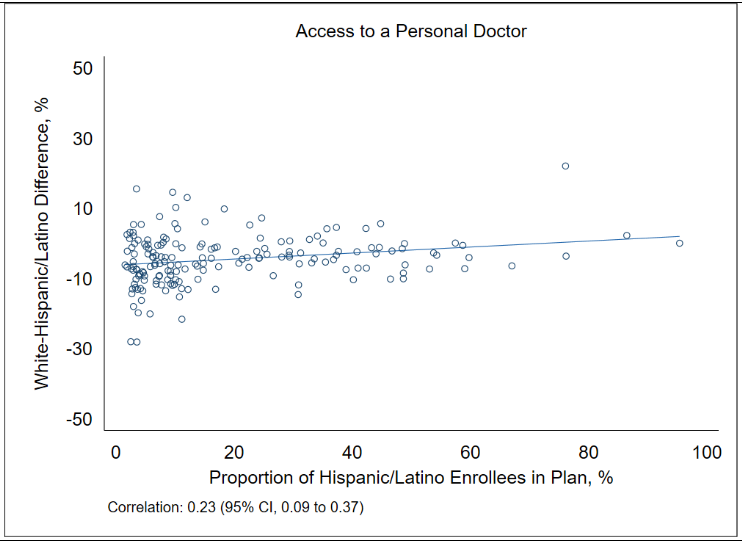

Panel C: Timely Access to Checkup or Routine Care

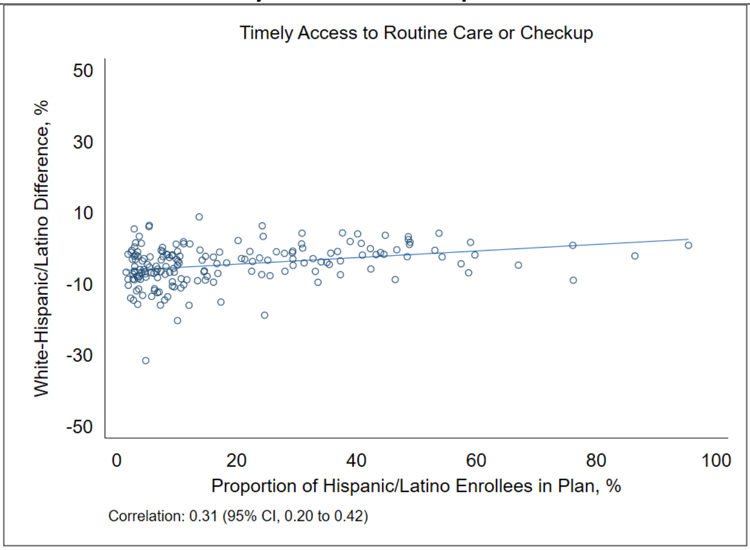

Panel D: Timely Access to Specialty Care

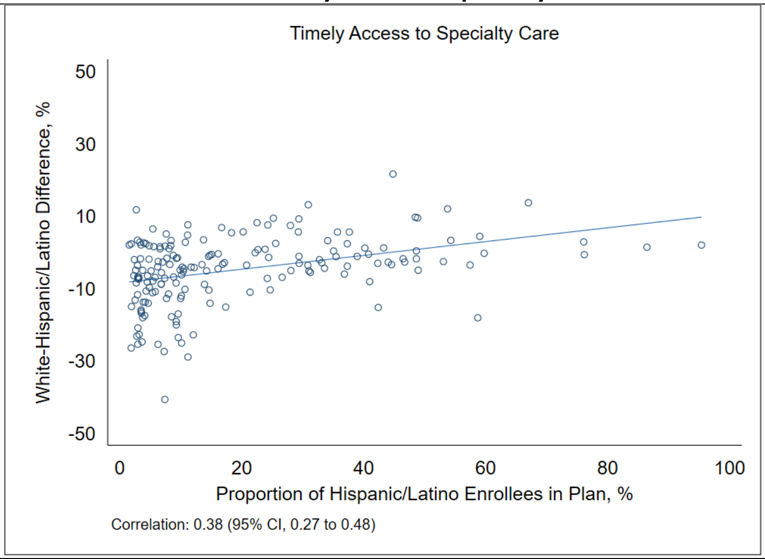

Source: Authors' analyses of National Committee on Quality Assurance (NCQA) Adult Medicaid Consumer Assessment of Healthcare Providers and Systems (CAHPS) Health Plan Survey 5.0, 2014-2018

Note. Analyses are at the plan level. Correlation calculated using Pearson's correlation coefficient and 95% CI calculated by bootstrapping standard errors with 10,000 replications.

Appendix Exhibit 23. White-AANHPI Between-Plan Disparities

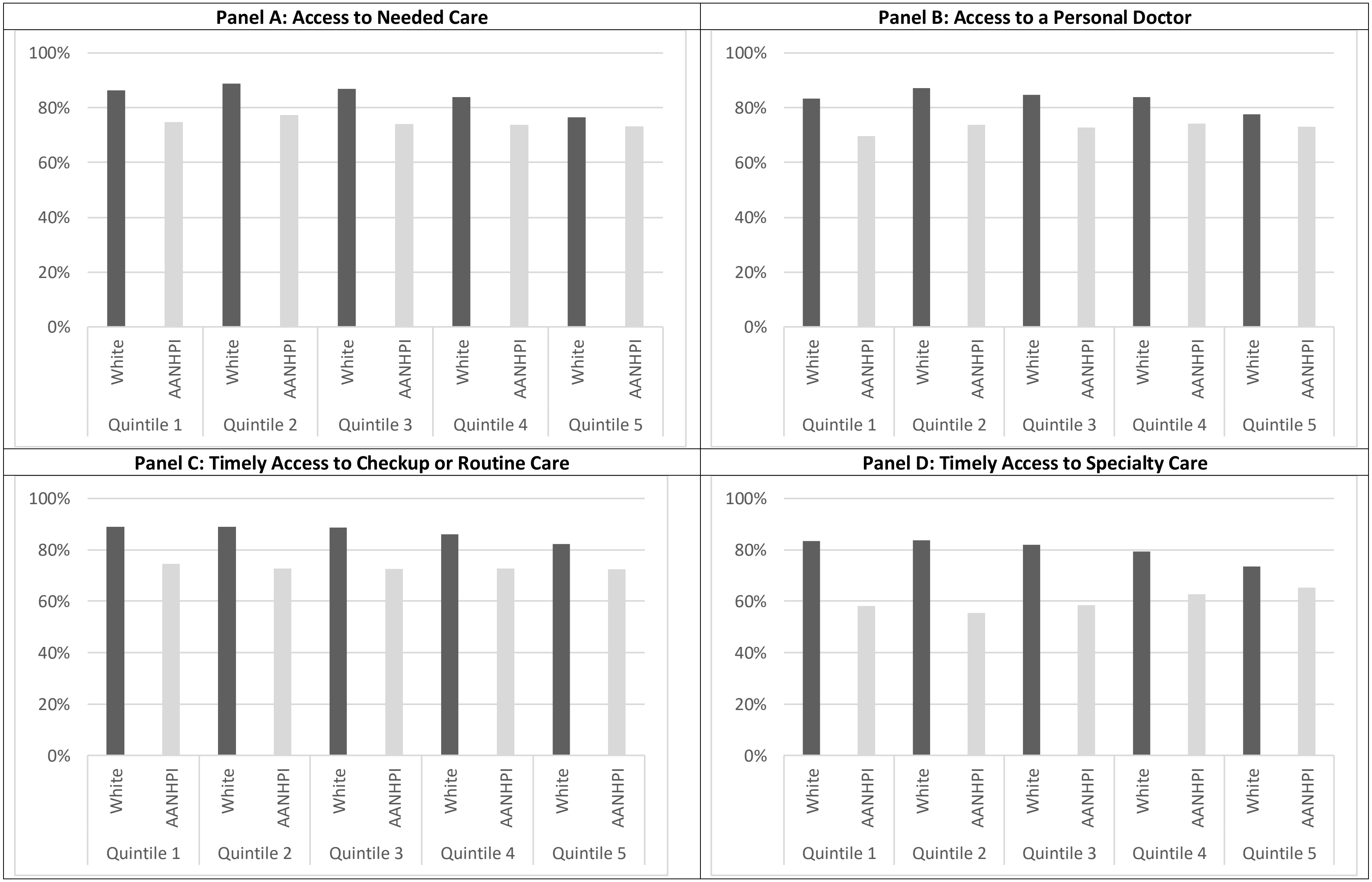

Source: Authors' analyses of National Committee on Quality Assurance (NCQA) Adult Medicaid Consumer Assessment of Healthcare Providers and Systems (CAHPS) Health Plan Survey 5.0, 2014-2018.

Notes. Quintiles reflect plan-level proportion of enrollees who are Asian American, Native Hawaiian, or Pacific Islander (AANHPI). Quintile 1 represents the 20% of plans with the lowest proportion of AANHPI enrollees and Quintile 5 represents the 20% of plans with the highest proportion of AANHPI enrollees.

Appendix Exhibit 24. Relationship Between Proportion of AANHPI Enrollees and White-AANHPI Disparities

Panel A: Access to Needed Care

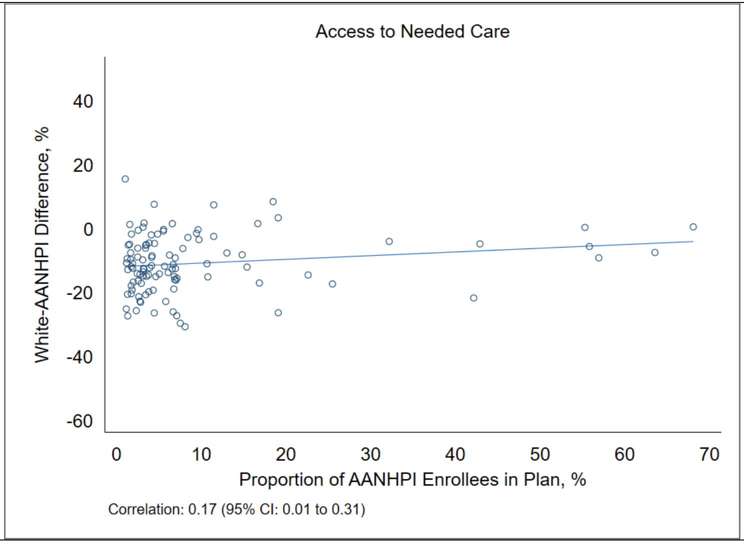

Panel B: Access to a Personal Doctor

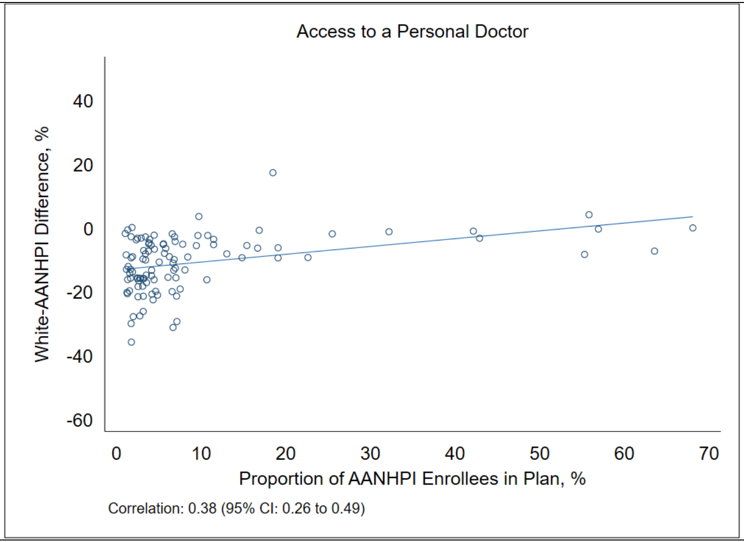

Panel C: Timely Access to Checkup or Routine Care

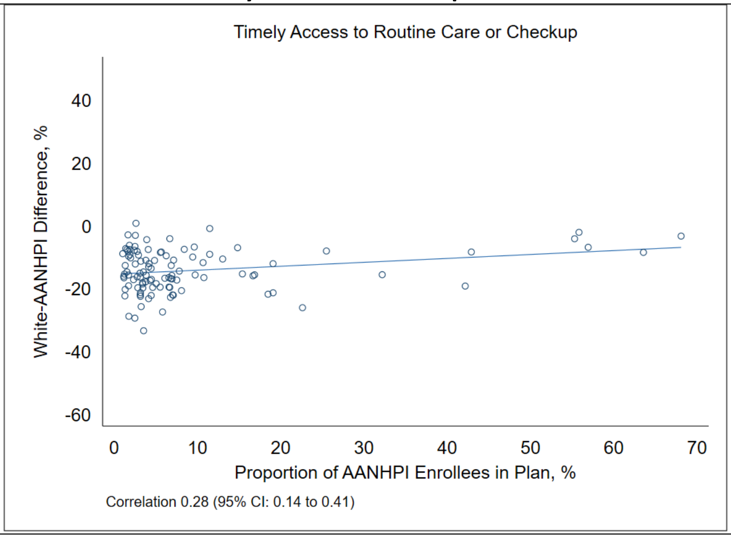

Panel D: Timely Access to Specialty Care

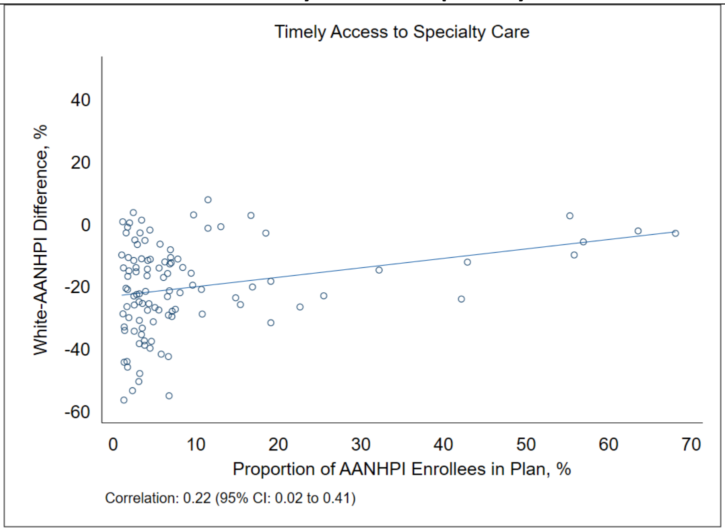

Source: Authors' analyses of National Committee on Quality Assurance (NCQA) Adult Medicaid Consumer Assessment of Healthcare Providers and Systems (CAHPS) Health Plan Survey 5.0, 2014-2018

Note. AANHPI refers to Asian American, Native Hawaiian or Pacific Islander. Analyses are at the plan level. Correlation calculated using Pearson's correlation coefficient and 95% CI calculated by bootstrapping standard errors with 10,000 replications
